# A web-based intervention for abused women: the New Zealand *isafe* randomised controlled trial protocol

**DOI:** 10.1186/s12889-015-1395-0

**Published:** 2015-01-31

**Authors:** Jane Koziol-McLain, Alain C Vandal, Shyamala Nada-Raja, Denise Wilson, Nancy E Glass, Karen B Eden, Christine McLean, Terry Dobbs, James Case

**Affiliations:** Centre for Interdisciplinary Trauma Research, Auckland University of Technology, Private Bag 92006, Auckland, 1142 New Zealand; Faculty of Health and Environmental Sciences, Auckland University of Technology, Auckland, New Zealand and Ko Awatea, Auckland, New Zealand; Department of Preventive and Social Medicine, University of Otago, Dunedin, New Zealand; Taupua Waiora Centre for Maori Health Research, Auckland University of Technology, Auckland, New Zealand; School of Nursing, Johns Hopkins University, Maryland, USA; Department of Medical Informatics & Clinical Epidemiology Department, Oregon Health & Science University, Oregon, USA; James Reed Case Consulting, Oregon, USA

**Keywords:** Partner abuse, Randomized controlled trial, Protocol, EHealth, Computer-assisted decision making, Internet, Safety, Mental health, Violence, Female

## Abstract

**Background:**

Intimate partner violence (IPV) and its associated negative mental health consequences are significant for women in New Zealand and internationally. One of the most widely recommended interventions is safety planning. However, few women experiencing violence access specialist services for safety planning. A safety decision aid, weighing the dangers of leaving or staying in an abusive relationship, gives women the opportunity to prioritise, plan and take action to increase safety for themselves and their children. This randomised controlled trial is testing the effectiveness of an innovative, interactive web-based safety decision aid. The trial is an international collaborative concurrent replication of a USA trial (IRIS study NCT01312103), regionalised for the Aotearoa New Zealand culture and offers fully automated online trial recruitment, eligibility screening and consent.

**Methods/Design:**

In a fully automated web-based trial (isafe) 340 abused women will be randomly assigned in equal numbers to a safety decision aid intervention or usual safety planning control website. Intervention components include: (a) safety priority setting, (b) danger assessment and (c) an individually tailored safety action plan. Self-reported outcome measures are collected at baseline and 3, 6, and 12-months post-baseline.

Primary outcomes are depression (measured by Center for Epidemiologic Studies Depression Scale, Revised) and IPV exposure (measured by Severity Violence Against Women Scale) at 12 months post-baseline. Secondary outcomes include PTSD, psychological abuse, decisional conflict, safety behaviors and danger in the relationship.

**Discussion:**

This trial will provide much-needed information on the potential relationships among safety planning, improved mental health, reduced violence as well as decreased decisional conflict related to safety in the abusive relationship. The novel web-based safety decision aid intervention may provide a cost-effective, easily accessed safety-planning resource that can be translated into clinical and community practice by multiple health disciplines and advocates. The trial will also provide information about how women in abusive relationships safely access safety information and resources through the Internet. Finally, the trial will inform other research teams on the feasibility and acceptability of fully automated recruitment, eligibility screening, consent and retention procedures.

**Trial registration:**

Trial registered on 03 July 2012 on the Australian New Zealand Clinical Trials Registry ACTRN12612000708853.

## Background

Intimate partner violence (IPV) is a widespread problem with significant negative health outcomes for survivors and their families [1-3]. Beyond the well-known negative physical health impacts, research consistently demonstrates a strong association between IPV and increased rates of depression, post-traumatic stress disorder (PTSD), substance abuse and suicide [4-6]. One of the most widely recommended interventions for abused women is safety planning [7-9]. The safety process involves a woman considering complex individual, cultural and community factors, such as financial needs and well-being of children. The challenge is to help women experiencing abuse to identify safety priorities and develop a personalised safety plan while considering staying or leaving an abusive relationship. In response to the challenge, USA researchers developed and are testing the first interactive, web-based safety decision aid for women experiencing IPV [10]. Preliminary findings suggest that women randomised to the safety decision aid reported less decisional conflict about their safety in the abusive intimate relationship after one use compared to women randomised to the usual safety planning condition [11]. While the USA-based IRIS study is the first trial to report on the evaluation of a decision aid for IPV survivors, the initial findings are consistent with a Cochrane systematic review that identified 115 trials testing health related decision aids [12]. The review reported that decision aids improve knowledge, create more accurate expectations of possible harms and benefits, increase active decision-making, and reduce the proportion of participants who report being undecided.

The New Zealand (isafe) research study is part of an international collaborative and concurrent replication of the USA IRIS trial (NCT01312103) testing the effectiveness of an interactive web-based safety decision aid in improving mental health and reducing IPV exposure. In the first phase of the New Zealand study, focus groups with service providers and women experiencing IPV informed regionalising the IRIS safety decision aid for the New Zealand context [13]. In addition to cultural tailoring [14], the New Zealand trial advances the IRIS study by offering women fully automated online trial recruitment, eligibility screening, consent and retention procedures.

## Methods/Design

### Trial design

The isafe trial design is a two-arm, parallel, randomised controlled trial to test the effectiveness of a web-based safety decision aid intervention on mental health and IPV exposure with abused women. The isafe trial in Aotearoa New Zealand involves a Māori Kaumatua (respected person who is a recognised elder) to advise and guide the researchers in the development and implementation of the trial to ensure processes are culturally responsive to Māori (New Zealand’s indigenous peoples) and to ensure the trial optimises Māori involvement. The trial protocol was developed to align with researcher agreed tikanga (principles) that included for example, ‘Respect and optimise the mana/status, tapu/safety and welfare of the wāhine/women, tamariki/children and whānau/family’. To increase trial access for women with a disability, the website addressed Web Content Accessibility Guidelines [15] and provided an audio option. The protocol is described according to the CONSORT-EHEALTH checklist [16].

Primary hypotheses are that: At 12 months post-baseline the intervention group (safety decision aid), in comparison to the control group (usual safety planning), will have: (1) improved mental health and (2) reduced IPV exposure. Secondary hypotheses are: At 3 and 6 months post-baseline the intervention group, in comparison to the control group, will have: (1*) improved mental health and (2*) reduced IPV exposure. At 3, 6 and 12 months post-baseline the intervention group, in comparison to the control group, will have: (3) increased safety-seeking behaviours and (4) less decisional conflict related to safety. Also, (5) increased benefit (improved mental health and reduced IPV exposure) from the intervention will occur under increased safety-seeking behaviours and less decisional-conflict at 3, 6 and 12 months. The effects on each outcome of the interaction between time and intervention arm, and of safety decision aid usage level, will form additional secondary hypotheses.

### Participants and recruitment

The target population is English-speaking adult women (≥16 years) residing in New Zealand who report current IPV. Current IPV is determined by a positive response to one or more of the following: (a) In the last 6 months, the woman has been hit, kicked, punched, choked (strangled) or otherwise physically hurt by the current partner; (b) In the last 6 months, the woman’s partner forced sexual activities or coerced her into sexual activities with threats; (c) In the last 6 months, the woman’s partner threatened to harm her physically; or (d) In the last 6 months, the woman has felt unsafe in the relationship.

Eligible women need to express comfort with their ability to access a safe and secure computer to login to the trial website. They also need access to a safe email, meaning they are the only one with password access to the account, to send and receive study-related information.

We conservatively seek to enrol an average of 43 women per quarter. This target is based on population-based 12-month partner violence prevalence rates estimated at 4 to 6% [2,17]; on calculated nationwide monthly averages [18] of 6,040 police domestic violence attendances and 4,125 crisis calls to Women’s Refuge; and the Recovery via Internet from Depression (RID) trial experience of 46 successful enrolments per quarter.

Women are recruited to the fully automated web-based study by registering on the trial site at www.isafe.aut.ac.nz. Strategies alerting women to the trial include referral to the site by community partners (specialist domestic violence agencies and police) and a range of social media advertisements which request volunteers for a confidential research study on safety in relationships. Such advertisements are made available on healthcare site-based TV, YouTube, Twitter and domestic violence and general social media websites. A free phone number is available for women to call with queries.

### Safety and security

The trial protocol was approved by the Auckland University of Technology Ethics Committee (AUTEC) in April, 2012. Safety protocols and research team training addressed safety assessment for women and their children, safe use of computers and internet, and IPV and suicidality resource referrals. Expecting a significant proportion of participants to have symptoms consistent with depression and suicidality [19], a message is included at the end of the depression scale (CESD-R) for all women that acknowledged ‘feeling down’, that ‘it’s important that you tell someone you trust how you are feeling’ and offers community-based referral information.

A data monitoring committee (DMC) meets 6 monthly during the trial. A DMC Charter outlines responsibility for safeguarding the interests of trial participants, assessing the safety and efficacy of the interventions during the trial, and for monitoring the overall conduct of the trial. Any unanticipated problems potentially related to isafe are reported to the DMC and AUTEC chairpersons.

### Procedures

Participants enrol in the study by accessing a secure New Zealand registration trial website (www.isafe.aut.ac.nz). The site provides a full description of the trial with potential risks and benefits. A participant is considered enrolled if they have visited the registration website and: met eligibility criteria (16 years or older, English language, current IPV), consented to participate, provided contact information, and been validated as a female resident in New Zealand. During registration, women are asked how they may be safely contacted and details for a contact person who would be able and willing to safely pass on a message to her should we lose connection with her during the 12-month follow up period.

With a fully automated online trial recruitment, a validation process was used to minimise the risk of fraudulent participant entry (such as duplicate entry). Validation is either automated (matching the consenting participant’s name and address against the New Zealand Electoral Roll file provided 09 May 2012) or manual validation. Manual validation is completed by a research team member conducting a logic check of participant information (such as birthday) against information gathered by Google and Face Book searching or alternatively by sending an e-mail requesting confirmation of electoral roll status from the participant. When unsure about a validation, research team members, with the PI, consult for a final decision.

Once enrolled, a woman is randomised by computer algorithm to either the intervention or control group and sent an e-mail with a username, password and URL access for either the intervention or control website. The woman has a 6 week window to enter the secure website and complete the baseline survey. Automated ‘reminder’ e-mails are sent during the 6 week window. Once a woman completes the baseline measures she is considered ‘accrued’. A woman who does not complete the baseline measures in the allocated time frame (within 6 weeks of enrolment) is considered ‘off study’.

Completion of study measures and either the safety decision aid or usual safety planning is estimated to take 45 to 60 minutes at each time point, a feasible time for participants to have web access and not be regarded as overly burdensome [13,20,21]. If women have to quit during a session they are able to log-in to the site at a later time. As a token of appreciation for participation, women provide a safe postal address to receive a $30 gift voucher at each measurement point (baseline, 3, 6 and 12 months).

To maintain contact with participants over the 12 month follow up period, automated emails are sent to participants at 1, 2, 4, 8 and 10 months. Retention mechanisms such as sending an email and or phoning participants at their provided ‘safe contact’ are initiated once women are ‘late’ for a follow up visit following safety protocols. This includes only using safe contact numbers/emails as provided by the participant. Logs are maintained by study personnel documenting phone and email contacts with participants, potential unanticipated events, and other issues affecting the trial (such as server interruptions).

### Safety decision aid intervention group

Women randomly assigned to the safety decision aid intervention group will be able to access the safety decision aid throughout the one-year study period via the secure password-protected trial website. The intervention includes three components: safety priority setting, danger assessment [22] and personalised safety action plan. The safety priority setting activity is based on a multi-criteria decision model [10]. The five criteria (priorities) listed in Table 1 were evaluated for the New Zealand context [13] with minor wording changes made to the descriptions. Women move a sliding bar toward the priority that is most important for all possible pairwise combinations. Through a series of matrix computations using the Analytic Hierarchy Process, the programme provides feedback to the woman, summarising her priorities.

**Table 1:** **Safety priority criteria**

**Priorities**	**Descriptions**
Having resources	Having housing and income.
Keeping my privacy	Issues in my relationship are not something I share with others.
My child’s well-being	Concerns for the well-being and safety of my child or children.
Feelings for my partner	Love or concern for my partner.
My concern for safety	Safety of myself, whānau, family and friends.

For the second component of the safety decision aid, women are asked to complete the Danger Assessment (DA) or Danger Assessment-Revised (for female same-sex relationships) and receive scored immediate feedback on their level of danger for severe or lethal violence in the intimate relationship, ranging from variable to extreme danger [23-27]. The DA includes a calendar to help women identify the frequency and severity of abuse and 19 lethality risk indicators related to the abusive partner’s behaviour and 1 item related to her risk of suicide. Once women receive feedback (based on summary score of the 19 items), they then have the opportunity to change their safety priorities. For example, if their danger level is higher than they expected, they may wish to make changes in their safety priorities.

The third and final safety decision aid intervention component is an interactive process using an underlying matrix of resources to help women develop an individually tailored action plan. The action plan lays out local, regional or national resources and tips about safety for the women and their children based on their safety priorities and DA scores. For example, women who screen positive for suicidality will receive messages with referral to their health provider as well as suicide prevention resources (Depression Helpline 0800 111 757 or Lifeline 0800 111 777). If women determine it is safe, they can print a copy of the personalised report with their action plan.

### Usual safety planning control group

Women randomly assigned to the control group will be able to access a list of contact details for key sources of support for partner violence throughout the one-year study period via the secure password-protected trial website. All control group women receive the same standardised list of resources and a standardised emergency safety plan, like those usually provided by domestic violence advocates, national hotlines or websites. Resources are not individualised to the safety needs of the woman as they do not complete the safety priority setting activity nor do they complete the DA. They are not provided with individualised feedback for an action plan.

### Outcomes

The primary outcomes consist of

- Mental health primary outcome: depression measured by the *Center for Epidemiologic Studies Depression Scale, Revised* (CESD-R) [28,29]. The CESD-R includes 20 depressive symptoms reflective of the DSM-IV criteria for depression. Participants respond ‘how often you have felt this way in the past week or so’ selecting one of five response options from ‘not at all or less than 1 day’ to ‘nearly every day for two weeks’. The total CESD-R Score may range from 0 to 60 (based on a CESD style score; see http://cesd-r.com/cesdr/) with scores of at least 16 indicative of depression. The CESD-R correlates highly with the original CESD and has strong psychometric properties (e.g., Cronbach's α > 0.90) in community samples [28-30].
- IPV exposure primary outcome: *Severity of Violence Against Women Scale* (SVAWS) [31]*.* The 46-item scale includes threats of violence (19 items; score may range from 19 to 76), acts of violence (21 items) and sexual violence (6 items). Participants select the frequency of the acts from ‘never’ (1) to ‘many times (4). The referent time period for this study was the past 6 months (at baseline and 12 month follow up) and the past 3 months (at 3 and 6 month follow up). Previous research has demonstrated good internal reliability (α = 0.90, 0.93 and 0.84 for the three subscales respectively) [32]. Researchers commonly report the threats of violence score (possible range 19 to 76) and actual violence (possible range 27 to 108) [32,33].

Secondary outcomes include

(c) Mental health secondary outcomes: *PTSD Checklist, Civilian Version (PCL-C)* [34,35]*, Alcohol Use Disorder Identification Test (AUDIT)* [36,37]*, Drug Abuse Screening Tool (DAST-10)* [38]*;*
(d) IPV exposure secondary outcome: *Women’s Experiences with Battering (WEB)* [39];
(e) Decisional conflict secondary outcome: *Decisional Conflict Scale* [40]*;*
(f) Safety seeking behaviour secondary outcome: *Safety Checklist* [41,42];

(g) Other secondary outcomes: *Danger Assessment or Danger Assessment – Revised (*for same sex couples) [23-26]*;* brief gambling screen [43,44]; and relationship intention.

### Randomisation and blinding

Computerised randomisation is based on a minimisation scheme which is a form of covariate adaptive randomisation and has the advantage of eliminating expected covariate imbalance. Two stratification factors (severity of violence and women with children) and 2 random factors each with 2 equiprobable levels are used to achieve the minimisation. Severity of violence factor based on one positive response to the current IPV eligibility items versus two or more. The children factor is based on the woman having one or more children versus none. All New Zealand trial investigators and team members are blinded to group assignment, with the exception of the data manager (JC) and trial statistician (ACV), who are responsible for the production of data monitoring reports.

### Statistical analysis

Descriptive statistics (including standard errors) for baseline outcome values and demographics will be produced by study arm. Additionally, we will monitor and collect information on women’s use of the website by both control and intervention participants to evaluate which information is accessed, skipped, or accessed over time.

The primary efficacy analyses will test Hypotheses 1 and 2 with outcomes (a) and (b) respectively. Secondary efficacy analyses will test Hypotheses 1 and 1* with outcomes (a) and (c); hypotheses 2 and 2* with outcomes (b) and (d); and hypotheses 3 and 4 with outcomes (e) and (f) respectively. All efficacy analyses will proceed on the basis of an assumed normal distribution for the residuals of the outcomes after regressing on the intervention arm and the baseline value. The underlying normality of the data will be assessed visually and using the omnibus *K*^*2*^ test [45]. Should non-normality be concluded, adequate alternative generalised linear models will be sought first, and appropriate transformations will be considered second. This evaluation will occur as part of the blind review. The data will be analysed using linear mixed models, wherein normally distributed random intercepts will be ascribed to each participant and an appropriate covariance structure selected for longitudinal measurements. All repeated measures data for an outcome, if available, will be included in the model, and appropriate contrasts estimated and tested to address the specific hypotheses.

In further secondary analyses, the effect of the intervention will be assessed in interaction with time. In addition, among the intervention group, we will examine the relationship between the number of times or “dose” the safety decision aid was used during the year and change from baseline to 12 months in the outcomes of safety seeking behaviours and exposure to violence using multiple regression. These analyses will proceed according to the same modelling framework as other efficacy analyses.

We will conduct planned subgroup analyses for each of hypotheses 1, 1*, 2, 2*, 3 and 4 and their associated outcomes. We will examine race/ethnicity, rural versus urban residence, and access to formal services as possible moderators of the effect of the intervention. Subgroup analyses will be conducted by considering the interaction between each subgroup and the intervention arm.

Hypothesis 5 will be tested by fitting a structural equations model with intervention as exogenous variable and safety-seeking behaviour and decision-making scores as endogenous, or mediation, variables to explain depression and violence scores (along with intervention arm). The repeated measures character of the data will be integrated in the model by including intercepts and mediating effects as participant-level random effects. This approach corresponds to the 1 → 1 → 1 lower level mediation model in Bauer et al. [46], who also provide a method to fit such a model with standard linear mixed modelling software. The structural coefficient estimates associated with safety-seeking and decision-making, as well as the intervention, and their 95% confidence intervals will be reported.

Bias may arise in the analysis if the intervention is effective and dropouts (women lost to follow up) are nonignorable (NI), that is, dependent upon the values of unobserved outcomes [47,48]. We will model the outcomes simultaneously with the dropout process using pattern-mixture latent-class models for Hypotheses 1–4, 1* and 2* [49,50]. This approach has been shown to reduce bias considerably in settings including mental health studies [50,51], and to be implementable using conventional statistical software such as SAS v.9.4 [51]. An attempt at extending the pattern-mixture latent class approach to the analysis of Hypothesis 5 will be made. Results from the joint outcome-NI dropout models will be presented along with the secondary analyses.

A blind review will be carried out at the end of follow-up to assess normality of residuals (excluding the treatment arm as regressor) and determine the best family or transformation to use if normality is rejected; to assess the appropriateness of the missing value strategies; to identify covariates that may improve efficiency or allay bias in case of imbalance; to identify an appropriate covariance structure for the repeated longitudinal measures; and to determine whether efficiency gains can be made by accounting for the covariance between the primary outcomes in the primary analyses.

Primary analyses will be conducted in an intention-to-treat analysis set. Secondary analyses of primary outcomes will also be conducted in a per protocol analysis set. Unmodified p-values will be reported for all tests, but inference for primary analyses will be based on False Discovery Rate controlled p-value thresholds accounting for the two outcomes. The nominal level for significance testing will be 5% against two-sided alternatives. All estimates of intervention effect will be reported as point estimates and 95% confidence intervals.

### Sample size

We estimate the required recruitment goal at 340 women. This figure is based on a need for 113 complete assessments per arm, accounting for an upper limit of 35% attrition by 12 months, based on rates from previous web-based studies (e.g., the RID trial, see http://www.otago.ac.nz/rid/). The estimated recruitment rate (43 women per quarter) will achieve the desired sample size over 36-month accrual period.

In regard to CESD-R: existing literature [28,52] is consistent with a correlation between baseline and 12-month CES-D/CESD-R measurements of about 0.5, consistent with a value of 0.47 deduced from data from a recent NZ trial [19]. Based on these data, we estimate that the planned study will have 80% power to detect a 30% decrease in the CESD-R depression score, at a 5% nominal confidence level Bonferroni-corrected for the two primary hypotheses. Based on these same data, this reduction represents an estimated relative decrease of 30.1% in the number or women at or above the cut-off of 16 for CESD-R, indicating depression. The detectable score reduction also corresponds to an effect size of 0.40 based on our data. (There is no established minimum clinically important difference for CESD-R).

In regard to SVAWS: we obtained an estimate of 29.2 for the standard deviation of the overall SVAWS, using pooled estimates of subscores from an international IPV study [53] and inferring subscore correlations from another [54]. The latter study also yielded an estimated correlation for the SVAWS at baseline and 12 months of 0.32. These indicate that the proposed study will have power 80% to detect a difference of −11.4 in IPV exposure, corresponding to an effect size of 0.39, at a Bonferroni-corrected nominal 5% significance level. We note that the Bonferroni correction is conservative with respect to False Discovery Rate control, and that gains in power are expected from the use of the repeated measures in the analysis.

## Discussion

This trial will provide information on the potential relationships among safety planning, reduction of decisional conflict, exposure to violence and mental health. The novel intervention used in this trial and its automated web-based delivery may set a new standard for safety planning that includes risk assessment, priority setting for decision making and creation of a personalised safety action plan. The web-based safety decision intervention may provide a cost-effective, evidence-based safety-planning tool that could be translated into practice by multiple health disciplines and advocates. The trial will also provide information about women in abusive relationships safely accessing the web and the experience of fully automated recruitment and retention processes.

## Trial status

Active follow up.
